# Annotations

**Published:** 1893-11-04

**Authors:** 


### ANNOTATIONS.

### Indian Oculists, British Jurors, and the Medical Council.

THE Indian Oculists have been acquitted. The law and not the facts was too strong for the jury. The twelve good men and true spent two hours in consultation after the Common Serjeant's summing up, and then returned to the Court with a legal verdict of acquittal, but with the following rider: "We very deeply deplore," said the foreman of the jury, speaking in the name of all the jurors, "that there exists at present no criminal law to prevent persons with such gross ignorance as the defendants from practising medical surgery." Commenting upon this the *Times* says: "If the Common Serjeant's reading is right, and if it is true that nothing except the death of a patient renders the quack doctor amenable to the criminal law, the sooner the Home Secretary looks into the matter the better." That we quite agree with. But there are other persons to whom the public and the medical profession have a rightful claim to look even more than to the Home Secretary. The Home Secretary has a thousand other things on hand beside the regulation of medical practice. But where is the General Medical Council? Where is the Royal College of Physicians? The London and Counties Medical Protection Society, a young Association consisting largely of family practitioners, has shown infinitely more public spirit than either of those more highly-placed and honoured organisations. The trial has rendered a great service to the public. It has shown that, short of killing his victim, any ignorant pretender may maltreat her Majesty's lieges for his own profit with absolute impunity. Is this the kind of protection which the General Medical Council was created to secure for the public? The Council seems to think ten times more of its own dignity than it does of the public safety. Dignity! What kind of dignity is it that pleases itself with voluminous talk and leaves its appointed work undone? There is no dignity in that. If the General Medical Council would draw up some practical measures for the public safety, and get them placed on the Statute Book, it would prove itself to be possessed of real dignity—the dignity of intellectual comprehensiveness and of practical utility in the state.

### Evening Out-patient Departments.

"A LIFE GOVERNOR" advocates in the *Globe* the opening of hospitals for out-patients after the work of the day is over. This is an old question, and deserves the most careful consideration on the part of hospital managers. Perhaps the whole problem might be solved in a single sentence if managers were sure that no persons would attend in the evening except those who have a real claim upon hospital aid, and who cannot, without serious loss, attend at any other time. If this point were absolutely settled, hospitals might make an effort to open their doors for an hour or two in the evening for the limited number of persons who would be found to fulfil the requisite conditions of need and desert. But, failing the possibility of this, one thing is very obvious at the outset, namely, that greatly as the out-patient departments of hospitals are now abused, they would be abused infinitely more if they were thrown indiscriminately open to all the industrial world at the very hour when all the industrial world has nothing particularly to do. The number of patients would probably increase fourfold, and the triviality of the cases would be in the direct ratio of the patients' numbers. Let any one who is in doubt on the subject try the step, as the writer has done, and very much to his own injury. What we would suggest is the absolute closure of all out-patient departments for a period of, say, six months, with a view to their re-organisation on a modern and rational basis. It must be remembered that doctors, though anxious for hospital appointments, give far more than a *quid pro quo* for those appointments. It is imperative that they should be considered. To conduct hospitals without due consideration for their honorary physicians and surgeons would be to drive away the best to a certainty, and to retain only the indifferent. What gain would that be to the poor, or, indeed, to anybody else? Our hospital system, like our political, has its defects and irregularities which appeal strongly to the ardent reformer. But it is very much easier to point out a wart on the face than to permanently cure it by the first rough and ready method which comes to hand.

### Is Alcohol a Food?

THE important question, "Is alcohol a food?" was answered by Dr. David Cerna in a paper read before the Pan-American Medical Congress, whose sittings have just been concluded. Dr. Cerna differs in his judgment from certain other physicians of weight and name who have discussed the question, in that he decides definitely that alcohol is undoubtedly a food. This conclusion he founds upon the facts of experience, observation, and experimentation, the only kinds of facts upon which rational conclusions can be based. It is impossible in the space at our disposal to give even a full summary of Dr. Cerna's positive affirmations, but a selection of those which are of the most obvious practical importance will be read with interest. Among other things, Dr. Cerna affirms that "alcohol in small amounts excites, in large amounts depresses the peripheral and sensory nerves," and that "excessive quantities cause degeneration of the axis cylinders of nerve fibres." With regard to the effect of alcohol upon the thought process, it is affirmed that "in small amounts the drug stimulates the cerebral functions, afterwards depressing, and finally abolishing them"; in other words, a little alcohol makes a man lively, whilst a great deal makes him "dead drunk." "Small doses of alcohol produce increased rapidity of the cardiac beat; large quantities a depression of the same." It is affirmed that "the drug kills by failure of the respiration." Physiologists of course know what is here meant, but the *profanum vulgus* will be apt to say that people die when the breath stops, even if they happen to have been teetotallers. Alcohol "lessens the excretion of tissue waste both in health and disease," says Dr. Cerna, therein corroborating the view of the late Sir Robert Christison. "In moderate amounts alcohol aids the digestive processes," we are told. Finally, "alcohol is a conservator of tissue, a generator of vital force, and may therefore be considered a food." It cannot be said that Dr. Cerna has added anything to the sum of knowledge on the subject of alcohol. The importance of his contribution consists in the fact that after careful and competent consideration of the whole question from every standpoint, he himself is personally convinced that alcohol is a real food.

